# Endocytosis of Nanomedicines: The Case of Glycopeptide Engineered PLGA Nanoparticles

**DOI:** 10.3390/pharmaceutics7020074

**Published:** 2015-06-19

**Authors:** Antonietta Vilella, Barbara Ruozi, Daniela Belletti, Francesca Pederzoli, Marianna Galliani, Valentina Semeghini, Flavio Forni, Michele Zoli, Maria Angela Vandelli, Giovanni Tosi

**Affiliations:** 1 Department of Biomedical, Metabolic and Neural Sciences, University of Modena and Reggio Emilia, Modena 41124, Italy; E-Mails: antonietta.vilella@unimore.it (A.V.); vsvalesuzz@gmail.com (V.S.); 2 Nanomedicine Group, Te.Far.T.I. Center, Department of Life Sciences, University of Modena and Reggio Emilia, Modena 41124, Italy; E-Mails: barbara.ruozi@unimore.it (B.R.); daniela.belletti@unimore.it (D.B.); francesca.pederzoli@gmail.com (F.P.); marianna.galliani@gmail.com (M.G.); forni.flavio@unimore.it (F.F.); zoli.michele@unimore.it (M.Z.); vandelli.mariaangela@unimore.it (M.A.V.)

**Keywords:** nanomedicine, blood brain barrier, neuron, endocytosis, cellular uptake

## Abstract

The success of nanomedicine as a new strategy for drug delivery and targeting prompted the interest in developing approaches toward basic and clinical neuroscience. Despite enormous advances on brain research, central nervous system (CNS) disorders remain the world’s leading cause of disability, in part due to the inability of the majority of drugs to reach the brain parenchyma. Many attempts to use nanomedicines as CNS drug delivery systems (DDS) were made; among the various non-invasive approaches, nanoparticulate carriers and, particularly, polymeric nanoparticles (NPs) seem to be the most interesting strategies. In particular, the ability of poly-lactide-co-glycolide NPs (PLGA-NPs) specifically engineered with a glycopeptide (g7), conferring to NPs’ ability to cross the blood brain barrier (BBB) in rodents at a concentration of up to 10% of the injected dose, was demonstrated in previous studies using different routes of administrations. Most of the evidence on NP uptake mechanisms reported in the literature about intracellular pathways and processes of cell entry is based on *in vitro* studies. Therefore, beside the particular attention devoted to increasing the knowledge of the rate of *in vivo* BBB crossing of nanocarriers, the subsequent exocytosis in the brain compartments, their fate and trafficking in the brain surely represent major topics in this field.

## 1. Introduction

### 1.1. Nanomedicine and Blood Brain Barrier

The same mechanism that protects the brain against intrusive chemicals and exogenous toxic agents can also frustrate therapeutic interventions. With the aim of effectively delivering a drug to the brain for treating specific neurological disorders (*i.e.*, meningitis, encephalitis, degenerative diseases like Alzheimer’s, and Parkinson’s disease and tumors as glioblastoma), one of the first issues to be addressed is to overcome the blood brain barrier (BBB), which may or may not maintain its integrity or permeability depending on the degree of pathology.

An ideal approach for the delivery of therapeutic agents across the BBB should be based on biodegradable, biocompatible carriers featuring the absence of toxicity to the barrier and producing the highest selectivity in therapeutic outcomes. Therefore, systemic delivery should be: (i) made of “safe” materials, as polymers or lipids; (ii) tailored for controlled/modified release of loaded drug; (iii) targeted to the BBB; (iv) to the site of target action in the brain; (v) able to allow the loaded drug to exert pharmacological action within the brain. To reach these goals, the transport of the drugs across the BBB should be appropriately planned and designed. In particular, proper evaluation of the main advantages of drug delivery systems in terms of ability in controlling the release of loaded drugs (*i.e.*, polymer-based carriers can ameliorate drug release kinetics with respect to lipid based ones), in being more biocompatible and able to interact with membranes (*i.e.*, lipid based systems can interact with a preferential modality or even are considered as bio-safe at a high degree) or in terms of stability in bloodstream (*i.e.*, polymer-based and lipid-based should be planned to avoid elimination/metabolism or even de-activation by Reticulo-Endothelial System).

To circumvent the multitude of barriers inhibiting CNS penetration by potential therapeutic agents, numerous drug delivery strategies (DDS) were developed [1,2]. These strategies fall into one or more of the following three categories: (i) chemical approach (lipophilic analogs, prodrugs); (ii) temporary disruption of the BBB (an invasive strategy for enhanced CNS drug delivery involving the systemic administration of drugs in conjunction with transient BBB disruption (BBBD)); (iii) alternative approaches for drug delivery as a molecular Trojan horse.

In this view, this strategy (“Trojan horse”) has been widely applied for macromolecules themselves, allowing them to be selectively targeted to a desired site of action in their inactive form and, only in the correct place, to be activated. Thus, from the conceptual point of view, the Trojan horse approach for nanocarriers is quite different, as it could be referred to as the “trick” of simulating and mainly exploiting natural/endogenous pathways for BBB crossing by non-self, but properly engineered, nanocarriers.

Despite advances in rational CNS drug design and BBBD, many potentially efficacious drug molecules still cannot penetrate into the brain parenchyma at therapeutic concentrations.

A winning strategy might consist in mimicking strategies used by the endothelial cells of BBB to exchange nutrients between CNS and blood in physiological conditions: the so called molecular Trojan horse. Briefly, a specific ligand can be bound onto the nanocarrier surface in order to enhance the CNS delivery [3,4,5,6,7,8,9,10].

In this paper, we analyze the case of a specific peptidic ligand (glycosylated hepta-peptide, called g7), which was demonstrated to be able to drive polymeric nanoparticles across the Blood Brain Barrier *in vivo* and with ability of interacting with neuronal cells (neurons mainly) with proper evaluations *in vitro* on cell cultures.

We think that this example, referring to one single kind of NPs which were analyzed from several points of view, and by several independent experiments, could be useful to plan future protocols and approaches for evaluation of the potentiality, the role and the fate of nanocarriers aimed to target the Central Nervous System.

### 1.2. Endocytosis of Nanomedicine

Two main transport routes have been exploited to actively target and trigger BBB crossing: carrier-mediated transport (CMT) and receptor-mediated endocytosis (RME).

CMT is a form of active or passive diffusion, depending on the context, and accounts for the unidirectional transport of molecules from the blood to the brain. The transporters are responsible for the brain delivery of substrates such as d-glucose (GLUT1 glucose transporter), large neutral amino acids (the LAT1 large neutral amino acid transporter, the CAT1 cationic amino acid transporter), carboxylic acids (the MCT1 monocarboxylic acid transporter) and nucleosides (the CNT2 nucleoside transporter) into the brain. CMT can be used for active targeting of CNS. From a general point of view, nutrients or their analogues, for which specific transporters exist on BBB endothelium, can be bound to the nanovector surface. By this strategy, the ligand transport into the CNS lumen leads also to the transport of the nanocarrier bearing the ligand on its surface. Into the brain tissue, the nanovectors can release their cargo by different mechanisms depending on the type of carrier used.

It should be clarified that the mechanism of BBB crossing needs to be completed in all the phases, meaning that the nanocarrier should be efficiently endocytosed, then submitted to transcytosis and require to be submitted to exocytosis. In some papers [11], it was demonstrated that if the linkage between the ligand and the receptors (*i.e.*, transferrin receptor) which trigger the BBB crossing is too heavy, the exocytosis process does not take place. This would mean that the nanocarrier will never be able to interact with brain cells, since it is still linked to the endothelial cell. This event is particularly evident in the case of the main receptors able to trigger BBB crossing, such as transferrin and insulin, and therefore, the use of some antibodies with a high degree of affinity to these receptors or even the natural substrates of the receptors were not completely successful.

RME or clathrin-dependent endocytosis is a highly specific and energy mediated transport enabling eukaryotic cells to selectively uptake macromolecules as specific cargo. The BBB receptor-specific ligands have also been shown to be very effective in transporting endogenous peptides like insulin, insulin like growth factor-I, insulin like growth factor-II, transferrin, albumin, and opioid peptides, e.g., deltorphins, (d-penicillamine 2,5) enkephalin and deltorphin II [12,13,14,15]. That is why receptor-mediated drug delivery is also a promising approach for the release of therapeutic agents into neuronal cells, and tissues; nanocarriers conjugated to different types of ligands for cell surface receptors expressed on brain endothelial cells can accumulate and eventually be internalized by cells on the vascular side of the brain through the mechanism of RME. Several receptors were investigated, such as transferrin (Tnf), insulin, thiamine, apolipoprotein receptors, beyond some peptides. OX-26 and 8D3mAb (respectively, monoclonal antibody to rat and mouse transferrin receptor) conjugated nanocarriers were proven to deliver drug molecules or exogenous genes to the brain [16,17,18,19]. 83-14mAb, a murine monoclonal antibody to the human insulin receptor, coupled liposomes have successfully delivered mRNA, siRNA or plasmids into the rat brain [20,21,22]. Apolipoproteins B and E were also suggested to be mainly involved in the transport of polysorbate 80 coated nanoparticle-bound drugs into the CNS: absorption of apolipoproteins from the blood after injection of surfactant-coated nanoparticles is likely to mimic lipoprotein particles that could be taken up by the brain capillary endothelial cells via RME [23].

Another category of approaches for BBB crossing is based on peptides able to trigger BBB crossing by mediating endocytosis of peptides into the CNS. In particular, some categories of opioid peptides have been shown to penetrate the BBB and this crossing is improved when peptides are glycosylated as described for enkephalin analogs, vasopressin analog, deltorphin and dermorphin glycopeptide analog and other peptides [24,25]. Hence, in this paper, we described the use of a specific class of peptides, able to cross the BBB and therefore suitable for being ligands for the planning and creation of delivery systems for the CNS [26]. The synthetic opioid peptide MMP-2200 (H2N-l-Tyr-d-Thr-Gly-l-Phe-Leu-l-Ser-O-beta-d-lactose-CONH_2_) was considered the lead [27]. The Tyr present at the N-terminus of MMP-2200 was substituted with the Phe in order to avoid a potential opioid effect. This glycopeptide was conjugated with PLGA to prepare modified polymers able to form NPs able to cross BBB [28]. While un-modified PLGA NPs were not able to cross BBB, peptide-conjugated NPs (g7-NPs) were able to cross the BBB by a systemic route g7-NPs are able to bypass the hepatic uptake, and to reach the brain [26,29]. Studies were also performed to assess the ability of these g7-NPs to act as drug carriers [30]. Loperamide, an opioid drug not able to cross the BBB, was loaded into the NPs and administered through systemic administration in rats; the evidence of loperamide delivery into the brain was achieved by the hot plate test (nociception assay). Loperamide-loaded NPs (2.7 or 1.8 mg/kg) produced a high antinociceptive activity 240 min after their administration. The ability of g7-NPs in BBB crossing was also confirmed by *in vivo* evidence, in rodents and after different routes of administration (i.p., i.v., nasal, oral) [31]. In particular, evidence of BBB crossing pathways was obtained after systemic administration of g7-NPs in rodents, indicating that g7-peptide, due to its peculiar amphipathic character, was able to selectively promote endocytosis at BBB level and therefore mediate BBB translocation of g7-NPs to the CNS parenchyma [32].

### 1.3. Endocytosis Mechanism and Fate of NPs

Once the nanocarriers have crossed the BBB, a big issue is to understand their trafficking, their endocytosis at a single cell level and their preferential tropism.

In this view, a plethora of papers were published regarding the endocytosis of nanomedicine. As reported in a very complete review on this topic [33], nanomedicines can employ multiple pathways for cellular entry. As clearly evidenced, the role of particle size, the shape, the material composition, surface chemistry and/or charge for utilization of a selected pathway(s) may strongly impact on the mechanism of internalization. Moreover, these parameters should effect also cell type accumulation and the overall fate of a nanomedicine relating to the effect of cell type on the processing of nanomedicines and the nanomaterial–cell interactions on the processes of endocytosis, as well as the resulting cellular responses [33]. In particular, their surface characteristic could strongly impact on cellular entry through definitive endocytic route(s) which could vary from caveolae- or clathrin-mediated pathways.

Endocytosis of nanomedicine was deeply investigated, mainly *in vitro*, and with different outputs, leading to conflicting results [34]. The main issue is regarding the translatability of *in vitro* results to *in vivo* application and the reproducibility of the results.

Few papers in reality report the effective *in vivo* endocytosis mechanism of NPs. As reported in a pivotal review on this topic: “developing assays to study the complex process of endocytosis of nanomedicines *in vivo* remain a key challenge for the future success of this field” [33].

One of the few papers which deeply investigated the endocytosis hypothesis is related to the exact mechanisms behind intracellular delivery of therapeutic compounds, endocytosis and pore formation which are involved [35]. The authors analyzed ultrasound and microbubble targeted delivery of therapeutic compounds and conclude that an evoked transient pore formation is happening (as demonstrated by the influx of calcium ions with the contribution of endocytosis being dependent on molecular size). This study is pivotal in the future direction of a real analysis of the molecular events which take place in the formation of endocytotic vesicle and it could be transferred to other examples, even in the case of neurons.

Moreover, a deep debate is underway on the role and real value of *in vitro* experiments on endocytosis. Duncan and colleagues clearly demonstrated that the conditions of *in vitro* experiments (serum free, continuous incubation in cells and concentration of medium) are too far from *in vivo* environments [34]. Thus, a confirmation of any *in vitro* experiments with *in vivo* proofs must be obtained and the *in vitro* experiments should be planned accurately to mimic or at least to shorten the distance with *in vivo* condition. In fact, as reported “the substrate concentration, the kinetics of uptake, the time dependence of internalization and intracellular trafficking should be carefully considered when setting a protocol for *in vitro* studies in order to maximize relevance of the data obtained to the *in vivo*/clinical setting” [34].

Another big issue regards the healthy or diseased conditions of the target site on the uptake of nanomedicine: above the well-known debate on the role of inflamed or diseased BBB in the CNS targeting [36,37,38,39] the impact of the disease conditions in endocytosis routes is still unclear and needs a further clear assessment. In this view, the future direction of the research in this field is to verify that all *in vitro* cell models and *in vivo* disease models are properly validated with respect to their functional endocytosis and trafficking behavior. Thus, it becomes almost clear that the evaluation of possible failure or enhancement of the drug efficacy mediated by nanomedicine will not be linked only to pharmacological resistance or intrinsic causes of the drug, but also related to nanomedicine endocytosis, uptake and accumulation.

In this view, as reported previously, PLGA NPs endocytosis and uptake modified with g7 peptides were deeply investigated. This field of research represents a good example of *in vitro* and *in vivo* correlation with respect to experimental data. In fact, electron photomicrographs showed the ability only of g7-NPs to cross the BBB as evidenced by several endocytotic vesicles and macropinocytotic processes. In previous papers, we deeply analyzed the modality and mechanism of BBB crossing of g7-NPs in comparison with un-modified NPs: *in vivo* experiments on rodents clearly demonstrated that un-modified NPs (namely only made of the same polymer, PLGA, without any surface modification) were not able to be detected into the brain parenchyma, but only lying in the brain vessels [32].

Computational analysis on the conformation of the g7- and random-g7-NPs on the NPs surface was also performed. It showed a different conformation (linear *versus* globular), suggesting a different interaction with the BBB.

Moreover, a deep study on the most important parameters (internal angles, distances, internal and planar/antiperiplanar conformation angles) was conducted on the g7-peptide, demonstrating that only g7*-*peptide does assume the helix-like conformation proposed by Pauling [40], *i.e.*, the now called “Biousian Conformation”, already demonstrated for the native opioid peptide MMP-2200 [27].

The computational analysis showed the Biousian structure of the g7 peptide [25,27,41], while on the contrary, the random-g7 peptide showed a globular conformation, suggesting that this difference is pivotal to explain the BBB crossing and allows to hypothesize this as the mechanism of BBB crossing displayed by the g7-NPs.

In order to hypothesize the mechanism of BBB crossing, it is important to consider that g7 derived from the native opioid peptide MMP-2200 and its ability to cross the BBB could be connected to the same mechanism used by the β-endorphines. These peptides cross the BBB adopting a helical amphipathic conformation in presence of BBB luminal wall lipid bilayers for which they have a high affinity [41]. It was demonstrated that these opioid peptides display a high level of amphipathicity with two conflicting solubility states (water-soluble random coil conformation and another at water-membrane phase boundaries); this conformation allows the helix insertion into the biological bilayers, correlated to a membrane-membrane interaction producing a membrane curvature [42].

g7-NPs could cross the BBB with the same modality, but with proper consideration of the “solid nature” of the carriers. Rigid protein, with intrinsic curvature and bound to the membrane surface, stabilizes the membrane curvature, which is mediated by the insertion of amphipathic moieties or helices of proteins between the polar head-groups of lipid molecules [43].

The same event is connected to g7-NPs, with a polymeric matrix structure (rigid) and decorated with an amphiphatic glycopeptides (g7) having helix-like conformation. Therefore, they could behave as a rigid high MW protein, producing the BBB membrane curvature.

In previous papers [32], we clearly showed a real interaction of the g7-NPs to the surface of the BBB, confirming the involvement of the amphiphatic helices supposed by Polt *et al.* Moreover, after *in vivo* administration, we detected several ruffles, referable to macropinocytosis, were sometimes recognized when g7-NPs are near to the endothelial cells. It is well known that macropinocytosis, although not frequent at BBB level, is the major mechanism responsible for the BBB crossing by different pathogens [44,45,46] as well as by different nanoparticulate carriers.

Taken together, these lines of evidence suggested that g7-NPs BBB crossing is owing to multiple pathways, mainly membrane–membrane interaction and macropinocytosis-like mechanisms.

Thus, these NPs cross the BBB by multiple pathways, such as endocytosis and macropinocytosis, and these pathways could play a pivotal role also in function of the progression of some CNS diseases.

### 1.4. Trafficking inside the CNS

The trafficking inside the CNS parenchyma and the preferential accumulation of NPs into neuronal cells is a poorly investigated issue *in vitro* and *in vivo*. Although only few examples could be found in the literature [47,48,49], if NP and BBB crossing is a well-known topic, it seems that the destiny of NPs after crossing the BBB is not known. On the contrary, the real fate of NPs is linked to their cell accumulation, intercellular trafficking, endocytosis processes and intracellular pathways, and therefore adequate understanding and investigation should be developed.

Very recently, the cellular and intracellular destiny of g7-NPs after *in vivo* administration in mice (i.p.) was assessed [47,48]. In particular, as g7-NP distribution within the brain and their interaction with CNS cells need to be accurately assessed before they can be proposed for therapeutic use, *in vivo* administration of g7-NPs was demonstrated to lead to a region- and cell type-specific enrichment of NPs within the brain. g7-NPs can cross the BBB and target specific brain cell populations, suggesting that these NPs can be promising carriers for the treatment of neuropsychiatric and neurodegenerative diseases. Besides, as the fate of NPs once entered in the brain after crossing the BBB and taken up into neuronal cells is a neglected area of study, the possible mechanisms of a cell-to-cell transport of g7-NPs was investigated. *In vivo* experiments clearly showed that g7-NPs can be transported intra- and inter-cellularly within vesicles after vesicular internalization. Moreover, cell-to-cell transport is mediated by tunneling-nanotube (TNT)-like structures in cell lines and most interestingly in glial as well as neuronal cells *in vitro*. The transport is dependent on F-actin and can be increased by induction of TNT-like structures overexpressing M-Sec, a central factor and inducer of TNT formation.

### 1.5. Rab GTPase in Vesicular Transport

One group of proteins which are necessary for efficient intracellular membranous trafficking, including endocytosis, are Rab GTPases together with their effector proteins [50,51].

In their active states, Rab-GTP bound forms are distributed on the cytosolic face of specific membrane compartments where they regulate intracellular trafficking, including vesicle formation, motility, docking and fusion [52]. For example, Rab4, Rab5, and Rab11 are localized to early endosomes (EE) and serve different functions. In the endocytic pathway, both Rab4 and Rab5 proteins are associated with EE: Rab4 has been shown to be involved in the regulation of recycling from the EE and, in particular, Tfn receptor (TfR) recycling. Rab5, which is also on the plasma membrane, is important in the homotypic fusion between EE as well as in the transport to the early endosomal compartment (EEC) [53,54,55]. In addition, it seems to be also necessary for budding of vesicles from the plasma membrane and has been suggested to regulate transport beyond EEC [56]. An increase in the level of Rab5 stimulates endocytosis and expands the size of the EE but also accelerates recycling from the EE to the plasma membrane [54]; in contrast, overexpression of Rab4 increases the reflux of endocytic markers to the cell surface and induces the accumulation of endocytic tubes to a distinct recycling compartment [57]. Rab11, on the other hand, has been demonstrated to function in transport through the recycling compartment [58,59]. Two further examples of Rab proteins are localized to the late endosomal compartment: Rab7 has been shown to control late endocytic trafficking while Rab9 regulates transport from late endosomes to the trans Golgi network [60,61,62].

All types of Rab proteins are implicated in the regulation of distinct membrane traffic events in the cell.

### 1.6. How to Set up NP Trafficking

Another big issue is to start identifying a sort of protocol for screening and detecting the trafficking pathways of NPs. Therefore, we take advantage of the technology of NP labeling by means of polymer conjugation with dye [26,29,30,63,64,65]; with the use of Rhodamine conjugated PLGA, we thus aim to exploit confocal microscopy for clearly identifying, from a qualitative point of view, the cellular type/subtype in which NPs could be found. The set-up of this protocol is useful as the immunohistochemistry protocols are well established for cell markers (*i.e.*, neurons, astrocyte, interneurons) but not with labeled NPs and their interaction with cells. In this paper, we therefore provide hints for this kind of protocols. Beside this technological aspect, proper investigations on cell type accumulation by g7-NPs after systemic administration in animal model will be given.

Thus, in this paper, we tried to summarize some procedures and protocols which may help readers and researchers in performing experiments *in vivo* for detection of NPs (labeled) and for a clear assessment of the trafficking and accumulation of NPs in brain areas.

Moreover, some new insights on the fate of g7-NPs will be given, especially regarding interneurons and clathrin–caveolin mediated processes.

## 2. Case Analysis of g7-NPs

As for materials and methods used, the major problem in standardization of results is the lack of homogeneity in experimental procedures. Therefore, in order to speed up the process of translatability of nanomedicine for brain targeting into clinical application, we refer to standard protocols (see Supplementary Information) which were applied in all the experiments we performed. In this way, we hope to help new studies in the organization of the workflow and especially to be in the same line and direction to achieve a good comparison of results.

### 2.1. Nanoparticle Characterization and Confocal Protocols to Visualized NPs

g7-NPs were characterized in their chemical–physical and morphological features, showing dimensions always in the range of 150 nm (30 nm as standard deviation) and featured by a regular surface as shown in previous paper by SEM analysis (data not shown) [47,48,49].

In order to set up the visualization of NPs, a plethora of experiments were conducted to optimize confocal features and performance. One of the first issues which should be taken into great consideration is linked to set up all the signals and the potency of the laser emission. These data will notably impact the signal detection, the amplitude of the signal and, moreover, the NP visualization. Besides these parameters, an important topic is also related to the possible overlapping of signals.

In the case of g7-NPs labeled with Rhodamine, we set up a protocol for excitation/emission profiles clearly identifying NPs in comparison with other probes, featured by specific excitation/emission profiles. As reported in previous papers [64] after excitation at 514 nm, both PLGA and g7-PLGA showed the same emission profile, *i.e.*, a single emission peak close to 580 nm. Any mixture of PLGA and Rhod-PLGA did not show any overlap, showing absence of any interference in signals.

### 2.2. Nanoparticle Accumulation in Endocytic Structures: Clathrin and Caveoline Positive Vesicles

Clathrin mediated endocytosis represents the “classical route” of cellular entry, which is present and inherently active in all mammalian cells. For instance, it is responsible for uptake of essential nutrients like cholesterol carried into cells by low density lipoprotein (LDL) via the LDL receptor, or iron carried by Tfn via the TfR [33]. Recent studies reported that layered double hydroxide NP internalization in neurons is mediated by clathrin-dependent endocytosis and that NPs are targeted mainly to the clathrin endocytotic machinery [66,67]. As for layered double hydroxide NP [66], also g7-NP internalization is mediated by clathrin-dependent endocytosis in neurons [47,48]. To demonstrate this mechanism, we infused chlorpromazine hydrochloride or staurosporine, inhibitors of clathrin-dependent and caveolin dependent endocytosis, respectively, unilaterally within mouse hippocampus and, 15 min later, injected g7-NPs (300 ag/300 μL/mouse) peripherally. Infusion of the respective vehicles was performed into the contra-lateral hippocampus. As also occurred *in vitro*, we observed a marked decrease of g7-NP signal accumulation within hippocampal neurons in chlorpromazine-treated hippocampus but not in staurosporine-treated hippocampus. As demonstrated by confocal microscopy analysis g7-NPs strongly colocalized with clathrin related- but not with caveolin related-signal [47].

### 2.3. Determination of Positive g7-NP-Early Endosomes

In the literature on cellular uptake of NPs, the discussion is restricted to clathrin-mediated and caveolae-mediated endocytosis without any consideration of transport inside cells mediated by EE. EE, in fact, represents the first major sorting station of the endosomal-lysosomal pathway and the site of internalization and initial processing of different biological mediators and proteins. In neurons, the endosomal-lysosomal pathway performs a multiplicity of integral functions including internalization of nutrients and neurotrophic factors, degradation and recycling of receptors and integration of signaling information to relevant intracellular pathways.

**Figure 1:**
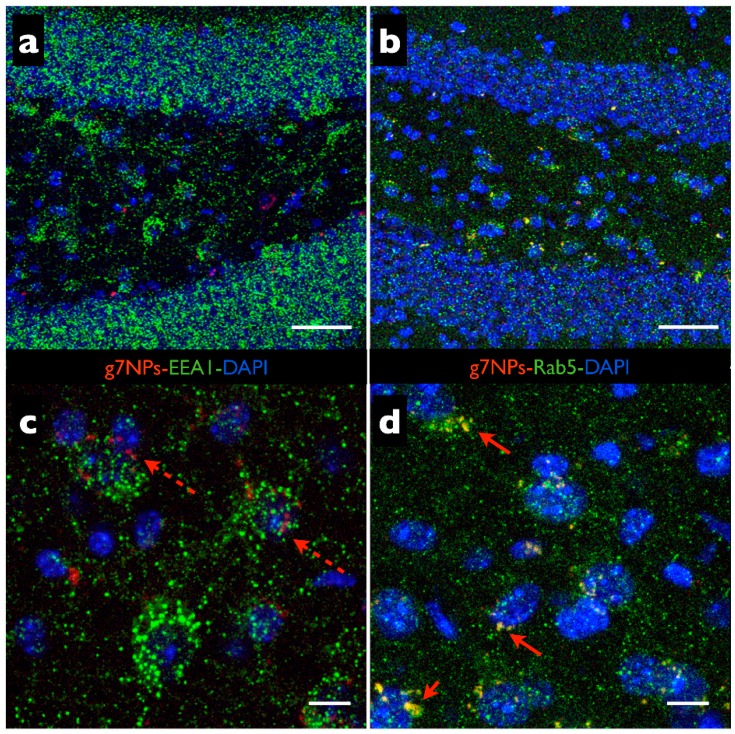
g7-NP uptake *in vivo*. (**a**–**d**) Confocal microscopy images of brain cryosections labelled with DAPI (blue), g7-NPs (red) and in green, rabbit anti-EEA1 (**a**,**c**) and rabbit anti Rab5 (**b**,**d**) from the hippocampal dentate gyrus of mice sacrificed 6 h after i.p. injection of g7-NPs. As shown in **c**, the dashed arrows indicate no overlapping between the red (g7-NP) and green (EEA1) signals; in **d**, arrows indicates a completely co-localization between red (g7-NP) and green signals (Rab5 ir) resulting in yellow labelling. Scale bar = 50 μm (**a**–**b**); 10 μm (**c**–**d**).

In particular, endosomal Rab5 localized in EE, is known to play important roles in regulating the various stages of endosomal trafficking and fusion and its function in neuronal endocytosis has received much recent attention. To better elucidate g7-NP transport inside cells, we tested the colocalization of g7-NPs with the clathrin-related Rab5 and caveola-related EEA1 early endosome markers. In Figure 1, we reported an example of impressive colocalization of g7-NPs and Rab5 signals in hippocampal interneurons but only minimal co-localization with EEA1 signals in the same cells.

This evidence, yet reported in previous papers [47,48], could be also connected to the accumulation of g7-NPs in presynaptic endosomes which are devoid of EEA1 [68]. Further experiments will be needed in order to better elucidate this aspect and to give new possible insights on the fate of NPs.

## 3. Commentary

The fate and destiny of NPs is still unclear. Unfortunately, the researchers in this field are still anchored to a very conservative way of thinking and planning of the experiments, which is based on the assessment of the phenomena (*i.e.*, presence or not inside the brain, pharmacological activity, *etc.*), but not the mechanisms. This approach was valid in past times, where nanomedicine was at its beginning and the knowledge on the mechanism of NP cell entry and BBB crossing was poor.

Nowadays, major awareness of the role of endocytosis of nanomedicines, of their trafficking once they have crossed the BBB and possible accumulation and tropism in detailed areas or even cell types is present. Therefore, much attention to these topics must be devoted with proper *in vivo* protocols, clearly assessing the behavior of nanomedicines.

In this view, we identified some pivotal points to be assessed in the evaluation of the potential of a nanocarrier for CNS drug delivery and targeting.

### 3.1. Technological Aspect

Significant effort should be made in the pharmaceutical nanotechnology field; this would mean that major attention must be devoted to the choice: (i) of the starting material (biodegradable, FDA approved, natural or synthetic) allowing to a “safe and ready-to-use nanocarrier”; (ii) of the technology used for nanocarrier production (safe, ready for scale up, avoidance of organic solvents or methodology for removal) allowing to a “ready-to-be produced nanocarrier” ; (iii) of the protocols for chemico-physical characterization (use of scanning electron microscopy, atomic force microscopy, *etc.*) which strongly impact the real assessment of a “standardized nanocarrier”.

### 3.2. In Vivo Experiments

*In vivo* analysis should be performed on healthy and diseased animal models, as the potential of nanocarrier (independently from the ligand used for BBB crossing) is strongly affected by the state of the BBB (increased or decreased permeability, alteration of tight junctions, altered transcytotic processes) and of the CNS (inflamed area, preferential uptake by cells, *etc.*).

### 3.3. In Vivo Sample Analysis

Beside the aspect of animal models and experiments, the methodology used for assessing proof-of-concept and even therapeutic advantages of nanocarrier approaches should be standardized and optimized. Even if the radio-labeling is one of the few methods in order to give qualitative and quantitative results in terms of CNS accumulation of both loaded drugs and nanocarriers, the use of other methods (*i.e.*, fluorescent labeled nanocarriers or drugs, pharmacological activity) needs a high level of procedural steps. If we will be able to find an optimized, transversal and globally applied protocol for NP detection (*i.e.*, confocal analysis followed by immunohistochemistry or use of labeled polymers with stable linkage with dye, *etc.*), all the results obtained in the laboratories of different research groups will be comparable.

By this new “wider” approach in nanomedicine investigation, the bench-to-bedside translatability of nanomedicines (from pre-clinical to clinical application) could be reached in shorter times as the validity of the experiments will be greatly improved.

## Supplementary Files

Supplementary File 1
