# Supplementary material for: Endocytosis of Nanomedicines: The Case of Glycopeptide Engineered PLGA Nanoparticles
Source: Pharmaceutics. 2015 Jun 19;7(2):74–89. doi: 10.3390/pharmaceutics7020074 (PMC4491652; doi:10.3390/pharmaceutics7020074)
Supplement: Supplementary File 1 [file pharmaceutics-07-00074-s001.pdf]

Supplementary Information

Procedures for CNS Targeting Experiment

Materials

All chemicals were obtained from Sigma–Aldrich (Milan, Italy). Poly(D,L-lactide-co-glycolide) (PLGA, RG502H, MW near to 11,000) was used as received from the manufacturer (Boehringer-Ingelheim, Ingelheim am Rhein, Germany). Gly-L-Phe-D-Thr-Gly-L-Phe-L-Leu-L-Ser(*O*-β-D-Glucose)-$CONH_2$ (g7) was prepared as previously described and conjugated with PLGA to obtain g7-PLGA [44–48]. The PLGA derivatization yields was confirmed by NMR, from the relative peak area of the signals at 7.2–7.5 ppm, corresponding to the aromatic protons of the Phe present and of the multiplet at 1.80–1.60 ppm, corresponding to the protons of the methyl groups of the polymer, respectively, and resulted to be in the range of 30–40 μmol peptide/g of polymer. PLGA conjugated with Rhodamine B piperazine amide (Sigma–Aldrich) (R-PLGA) was prepared as previously described [49]. A MilliQ water system (Millipore, Bedford, MA, USA), supplied with distilled water, provided high-purity water (18 MΩ). All the other chemicals were of analytical grade.

Preparation and Characterization of Rhodamine Labeled g7-NPs (g7-NPs)

Nanoparticles were prepared as already described before [23,28,29]. Briefly, to obtain labelled g7-NPs, a mixture composed by g7-PLGA (20 mg), R-PLGA (10 mg) and PLGA (70 mg) was dissolved in acetone (8 mL). The organic phase was then added dropwise into deionised water (25 mL) containing 100 mg of poloxamer 188 (Pluronic F68®, Sigma–Aldrich). After stirring at room temperature for 10 min, the organic solvent was removed at 30 °C under reduced pressure (10 mmHg). The final volume of the suspension was adjusted to 10 mL with deionised water. All the g7-NP preparations were purified by gel-filtration chromatography (Sepharose CL 4B gel, 160 mL, column 50 × 2 cm, Sigma–Aldrich), using water as the mobile phase and freeze-dried (Lyovac GT-2, Leybold-Heraeus, Hanau, Germany) without any cryoprotector. Before usage for *in vivo* experiments all the batches of NPs were characterized regarding their surface, chemico-physical, morphological properties as previously described [33].

Animal Procedures and Sample Preparation for Systemic Injection of g7-NPs

g7-NPs suspended in saline solution were injected i.p. in C57Bl6 mice. After 6 h animals were sacrificed. For histological processing, animals were anesthetized with chloral hydrate (400 mg/kg, i.p.). Intracardial perfusion was performed with 4% paraformaldehyde and 0.2% picric acid in Phosphate Buffered Saline (PBS) (70 mL/7 min) preceded by an infusion of 50 mL of 0.9% NaCl saline containing heparin sodium (5000 U/L). The brains were dissected out and postfixed in the same solution for 12 h, rinsed in 15% sucrose in PBS for approximately 12 h and then in a 30% sucrose in PBS for 1 day. The brains were frozen using dry ice, and coronal 50 μm thick sections series were cut at a cryotome, washed three times in cold 1× PBS and stored at −20 °C in a glycerol-PBS solution until use.

Immunohistochemistry of Brain Sections

Brain sections were processed for multiple immunofluorescence histochemistry according to the protocol previously described [33]. Briefly, after five washes with 1× PBS pH 7.4 for 10 min, blocking

was performed for 1 h at room temperature in a 1× PBS solution containing 0.1% Triton X100 and 1% bovine serum albumin (BSA). Incubation with primary antibodies diluted in 0.3% Triton X100, 1% normal serum (NS) and 1× PBS was performed overnight at 4 °C. After three washes in PBS/0.1% Triton X100, incubation with goat anti mouse Alexa488 (1:200) or goat anti rabbit Alexa488 (1:200) secondary antibody in 0.2% Triton X100, 1% NS and PBS was carried out for 90 min at RT. After washing three times with PBS for 10 min, brain sections were placed on gelatinised glass slides, dried and, after incubation with DAPI, mounted for confocal microscopy analysis.

Antibodies against early endosome antigen 1 (EEA1) were purchased from BD Biosciences (Buccinasco, Italy), Rab5 from Cell Signaling Technology (Danvers, MA, USA) clathrin from Abcam (Cambridge; UK) and Caveolin from Santa Cruz Biotechnology (Dallas, TX, USA).

Excitation-Emission Profiles

The emission profiles of the PLGA, g7-PLGA, Rhod-PLGA were evaluated by confocal microscopy (Confocal Microscope Leica DM IRE 2, Bannockburn, IL, USA; Leica Confocal System: scan head multiband 3 channels Leica TCS SP2 with AOBS, laser diode blu COH (405 nm/25 mW), Laser Ar (458 nm/5 mW) (476 nm/5 mW) (488 nm/20 mW) (496 nm/5 mW) (514 nm/20 mW), Laser HeNe (543 nm/1.2 mW), Laser HeNe (594 nm) (Orange), Laser HeNe (633 nm/102 mW). The emission profiles were assessed by applying confocal scansion over the variation of the wavelength, after excitation at 514 and 633 nm (Laser Ar and Laser HeNe, respectively) and recording the variation of the emission intensity.

Practically, an exact amount (5 mg) of the polymer (PLGA) or of one of the polymers conjugates (g7-PLGA, Rhod-PLGA) was solubilised in DCM (5 mL) and a drop (about 50 μL) of the organic solution was poured on a glass coverslip. After having removed the solvent at r.t., the confocal analysis was performed on the dry residue. The emission intensities of polymer and conjugates was registered over the variation of wavelength, using two different excitation lasers, in at least five regions of interest (ROIs). After excitation at 514 nm the emission profiles were detected from 560 to 810 nm every 10 nm, while after excitation at 633 nm the emission profiles were performed from 540 to 830 nm every 10 nm. To pointed out possible interferences between g7-PLGA and Rhod-PLGA, the excitation-emission profiles of a mixture of g7-PLGA and Rhod-PLGA at the same ratio used for the nanoparticle preparation (1:1.5, *w*:*w*) was carried out under the same experimental conditions above described.

© 2015 by the authors; licensee MDPI, Basel, Switzerland. This article is an open access article distributed under the terms and conditions of the Creative Commons Attribution license (http://creativecommons.org/licenses/by/4.0/).
